# Associations of adult physical activity with perceived safety and police-recorded crime: the Multi-ethnic Study of Atherosclerosis

**DOI:** 10.1186/1479-5868-9-146

**Published:** 2012-12-17

**Authors:** Kelly R Evenson, Richard Block, Ana V Diez Roux, Aileen P McGinn, Fang Wen, Daniel A Rodríguez

**Affiliations:** 1 Department of Epidemiology, Gillings School of Global Public Health, University of North Carolina - Chapel Hill, 137 East Franklin Street, Suite 306, Chapel Hill, NC, 27514, USA; 2 Department of Sociology, Loyola University, Chicago, IL, USA; 3 Center for Social Epidemiology and Population Health, University of Michigan, Ann Arbor, MI, USA; 4 Department of Epidemiology and Population Health, Albert Einstein College of Medicine, Bronx, NY, USA; 5 Department of City and Regional Planning, University of North Carolina, Chapel Hill, NC, USA

**Keywords:** Crime, Environment, Geographic Information Systems, Leisure activities, Physical activity, Safety, Social environment, Walking

## Abstract

**Background:**

Due to the inconsistent findings of prior studies, we explored the association of perceived safety and police-recorded crime measures with physical activity.

**Methods:**

The study included 818 Chicago participants of the Multiethnic Study of Atherosclerosis 45 to 84 years of age. Questionnaire-assessed physical activity included a) transport walking; b) leisure walking; and c) non-walking leisure activities. Perceived safety was assessed through an interviewer-administered questionnaire. Police-recorded crime was assessed through 2-year counts of selected crimes (total and outdoor incivilities, criminal offenses, homicides) per 1000 population. Associations were examined using generalized estimating equation logistic regression models.

**Results:**

Perceiving a safer neighborhood was positively associated with transport walking and perceiving lower violence was associated with leisure walking. Those in the lowest tertile of total or outdoor incivilities were more likely to report transport walking. Models with both perceived safety and police-recorded measures of crime as independent variables had superior fit for both transport walking and leisure walking outcomes. Neither perceived safety nor police-recorded measures of crime were associated with non-walking leisure activity.

**Conclusions:**

Perceived and police-recorded measures had independent associations with walking and both should be considered in assessing the impact of neighborhood crime on physical activity.

## Background

In the last two decades, physical activity researchers have moved beyond a focus on individual and psychosocial level determinants towards exploring how the environment relates to physical activity. Crime and perceived safety from crime have received special attention as environmental predictors of physical activity [1,2]. At least two research groups proposed theoretical conceptualizations of how physical activity is influenced by measures of safety. Loukaitou-Sideris and Eck [2] proposed that sociodemographic, psychological, and environmental factors all influence perceived risk and fear. The perception of risk and fear lead to constrained behavior, such as less physical activity. Foster and Giles-Corti [1] expanded further to propose both perceived (i.e., self-reported) and objective measures of safety influence outdoor physical activity. They also proposed other factors of influence, including individual, social environmental, and physical environmental factors. This framework combines perceived and objective measures of crime into one construct. However, it is not known if the more objective measures detect unmeasured aspects of perceived safety or alternatively if they affect physical activity even when a person is not aware or does not report it as a problem.

Prior studies relating perception of safety from crime to physical activity produced inconsistent findings, which could be due to the conceptualization and measurement of safety and physical activity, small sample sizes, differences across locations, and a limited number of individuals who perceived low safety in the samples studied [1,3,4]. Moreover, the impact of perception of crime on physical activity may be modified by other factors (i.e., gender, socioeconomic status, race/ethnicity, population density [4]) but many studies have lacked the statistical power to explore interactions.

The study of safety and crime can also benefit from objective measures of crime, since they are not dependent on self-report and may provide additional information on the context of the neighborhood. Few studies have explored the association of physical activity with objective measures of crime, including calls for service, reported crimes, or arrests [1]. It is important to acknowledge that these objective measures also have limitations. Calls-for-service are dependent on making an emergency call (such as to 911 in the United States (US)) and thus are underreported and may be differentially reported across neighborhoods [5]. Furthermore, classification of the call is made before an investigation is performed and an incident may be reported multiple times, hampering precise measurement of the crime. Reported crimes represent actual events, capture serious offenses well, and indicate that an investigation occurred, although differences in reporting across neighborhoods is a possibility [6-8]. Arrests represent a person, not a single event, and are not always linked to the crime type.

While evidence suggests that both self-reported and objective measures of crime have their limitations, using both measures may provide complementary information on neighborhood environmental characteristics that impact physical activity. However, few studies have explored the associations of crime and safety with physical activity using measures that both did and did not rely on self-report of crime [5,9-12]. Moreover, none of these studies reported on the combined associations of self-reported and objectively measured crime on physical activity.

We studied the relationship of both self-reported safety and police-recorded measures (e.g., using geographic information systems (GIS)) of crime on physical activity among adults from the Multi-ethnic Study of Atherosclerosis (MESA). Specifically, we examined associations of self-reported safety and police-recorded crime, separately and jointly, on transport walking, leisure walking, and non-walking leisure activities. A secondary aim was to explore whether the associations of perceived safety were modified by gender, education, and race/ethnicity. This study expanded prior work by including both perceived safety and police-recorded measures of crime, exploring both the independent and combined associations. This study also differentiated between several modes or types of physical activity, including transport walking, leisure walking, and non-walking leisure activities. We did this to explore whether the safety and crime measures might be differently associated with different modes of physical activity, particularly since correlates of transportation walking and leisure walking may differ. This addressed the call for greater specificity of measurement in environmentally motivated studies [1,13].

## Methods

### Source population

MESA (http://www.mesa-nhlbi.org) is a longitudinal study of adults ages 45 to 84 that aims to identify characteristics and risk factors for subclinical atherosclerosis at six study sites in the US [14]. The study was approved by the Institutional Review Boards at each site and all participants gave written informed consent. Participants were free of clinical cardiovascular disease at baseline and were recruited using a variety of population-based approaches. Telephone-based recruitment strategies were primarily employed, which resulted in high rates of inability to make initial contact with selected households, a high proportion of households refusing enumeration, and incomplete assessment of eligibility status. Among those screened and deemed eligible, the participation rate was approximately 60%. The baseline visit for the cohort, on which these analyses are based, occurred during August 2000 to August 2002. These analyses were restricted solely to the subset of participants at the Chicago, Illinois site residing in Cook County, since this was the only location where detailed police-recorded crime data were available.

### Physical activity measurement

A detailed interviewer-administered, semi-quantitative questionnaire adapted from the Cross-Cultural Activity Participation Study [15,16] was used to collect physical activity, including leisure, household, work, and transportation activities at baseline. The questionnaire was developed using extensive qualitative research [17] and physical activity records [15]. For each type of activity queried, participants were asked (a) whether they engaged in an activity during a typical week in the past month and (b) how many days per week and hours/minutes per day they did the activity. The activities were grouped into three mutually exclusive categories: (1) transport walking (e.g., walking to get places such as to the bus, car, work, or store); (2) leisure walking (e.g., leisure walking, pleasure, social reasons, walking during work breaks, walking the dog); and (3) non-walking leisure activities including (i) team sports (e.g., softball, volleyball, basketball, soccer), (ii) dual sports (e.g., tennis, racquetball, paddleball), (iii) individual activities (e.g., golf, bowling, yoga, tai-chi), and (iv) moderate- or heavy-effort conditioning activities (e.g., aerobics, bicycling, running, jogging, rowing, swimming, judo, karate).

Given unavoidable measurement error in reports of exact times of walking, the data were categorized rather than left continuous. Categories of self-reported physical activity data usually have better test-retest reliability than continuous measures, and allow for a way to manage data skewness [18]. As a result, the transport and leisure walking outcomes were categorized into tertiles using usual minutes per week. Non-walking leisure activities (including team sports, dual sports, individual activities, and moderate- or heavy-effort conditioning) could not be divided into tertiles due to the large percent of participants not engaging in them. Instead, they were categorized into three levels (zero, < median of nonzero data, and > =median of nonzero data).

### Perceived safety measurement

Participants answered questions about their neighborhood at the baseline examination. Neighborhood was defined for participants in the introduction to the questionnaire: “By neighborhood, we mean the area around where you live and around your house. It may include places you shop, religious or public institutions, or a local business district. It is the general area around your house where you might perform routine tasks, such as shopping, going to the park, or visiting with neighbors.” In particular, interviewers asked them, “How safe from crime do you consider your neighborhood to be?” The response options ranged on a 5-point scale from “very safe” (1) to “not at all safe” (5) and due to the distributions were grouped into three levels for modeling purposes (1 or 2, 3, 4 or 5). Participants were also asked if violence was a problem in their neighborhood, using a 4-point scale from “very serious problem” (1) to “not really a problem” (4). These four categories were grouped into 3 levels for modeling purposes: very or somewhat serious problem, minor problem, or not really a problem. Questions similar to these had acceptable test-retest reliability in other urban populations [19,20].

### Police-recorded crime measures

The Chicago Police Department categorizes incident reports of crimes by type and precise location. Addresses of crimes are recorded using look-up tables to ensure that each address is identifiable. In the case of an incident occurring at a location without an address (such as in the middle of a park), a pseudo address based on a predetermined Chicago grid is submitted. The result was that more than 99% of the incident addresses could be geocoded. These incident events were available to our research team aggregated to the census block group.

The types of crime we explored were categorized using similar definitions others have used [5], including incivilities (drugs, prostitution, vandalism) and criminal offenses (robbery, sexual assault, weapons). We also categorized homicides into its own category due to the severity of the incident. Using additional information provided on the location of crime, we created an indicator of whether or not the crime occurred in outdoor public places, such as occurring in the street, alley, sidewalk, park, or a vacant lot.

The MESA participant’s home address was geocoded and assigned to the US 2000 census block group in which the home was located. The same census data provided population information for each block group. Police-recorded crime measures for each participant’s census block group of residence was summarized over a 2-year period and normalized by the block group population into 2-year counts per 1000 population, by total and outdoor incivilities, criminal offenses, and homicides. The 2-year period included the year of the baseline visit and the preceding year. Incivilities and criminal offenses of the MESA participants were categorized into tertiles. Because of the small number of homicides, these were categorized as any (yes or no) and those areas with any homicides were further split at the median (< median of nonzero data or > = median for nonzero data).

### Measurement of other variables

Information on sociodemographic variables (age, gender, race/ethnicity, education, income, work status) were obtained at recruitment or at the baseline MESA examination. Participants self-reported how long they lived in their neighborhood and whether sidewalks were available there. For both questions, the neighborhood was defined as a 20-minute walk or about 1 mile from home. Distance to nearest public transportation was calculated as the Euclidean or straight-line distance to the closest bus line, commuter rail stop, or train stop using data from the Regional Transportation Authority (http://rtachicago.com/).

### Statistical analyses

A total of 1164 MESA Illinois participants completed the baseline interview and 823 resided in the city of Chicago. Of these, 5 were excluded due to being unable to geocode or match their geocodes to the crime data, leaving 818 participants available for analysis. Spearman correlations were used to compare each of the three continuous measures of normalized crimes occurring in a participant’s census block group, overall and among outdoor crimes. We explored agreement between perceived safety and police-recorded measures of crime before modeling, using Spearman correlations comparing perceived safety measures (as originally collected and not categorized) with continuous measures of the normalized police-recorded crime variables (total and outdoor incivilities, criminal offenses, and homicide).

Generalized estimating equation logistic regression models were used to estimate adjusted odds ratios between the 3-level measures of transport walking (tertiles), leisure walking (tertiles), and non-walking leisure activities (zero, < median of nonzero data, and > =median of nonzero data) with both perceived safety and police-recorded crime. Clustering by 2000 US census block group, as a proxy for neighborhood, was accounted for using an exchangeable correlation structure. First, we explored the perceived safety and police-recorded crime measures in separate models with each outcome. Next, after confirming lack of collinearity with the Spearman correlations, we modeled the perceived and police-recorded measures simultaneously. Using likelihood ratio tests we examined whether the addition of police-recorded crime measures improved the model fit to the fully adjusted model with perceived safety only. To explore the combined effects, we created variables using both perceived and police-recorded measures defined as follows: (1) safest group from both the perceived measure (agreement that neighborhood is safe) and police-recorded crime (lowest tertile), (2) least safe group from both the perceived measure (agreement that the neighborhood is unsafe) and police-recorded crime (highest tertile), and (3) all other participants. These combined measures were then examined in relation to each of the three physical activity outcomes.

For the statistical models that used perceived safety, we examined interactions with gender, education (high school or less, college, more than college), and race/ethnicity (non-Hispanic White, Other) using likelihood ratio tests. These models did not account for clustering. For the consistently statistically significant interactions (p < 0.10), we examined stratified models by the effect modifier. All analyses were conducted using SAS version 9.1.3 (Cary, NC).

## Results

### Description of area and sample

Characteristics of the sample are provided in Table 1. Participants reported living in their neighborhood on average 20.2 years (median 18.0 years, interquartile range (IQR) 8.0-30.0). Using GIS, the mean Euclidean distance from the participant’s home to the nearest public transportation (e.g., bus line, commuter rail stop, or train stop) was 0.06 miles (median 0.04 miles, IQR 0.02-0.07). Participants reported more time spent in transport walking (median 4 hours/week, IQR 1.8-7.0) than either leisure walking (median 2.0 hours/week, IQR 0–5.0) or non-walking leisure activities (median 1.0 hours/week, IQR 0–4.0 hours/week).

**Table 1:** Descriptive Characteristics of the Study Population (n = 818): The Multi-ethnic Study of Atherosclerosis

	**n**	**%**
Age group		
45-54	219	26.8
55-64	229	28.0
65-74	253	30.9
75-84	117	14.3
Gender		
Female	446	54.5
Male	372	45.5
Race/ethnicity		
Non-Hispanic White	462	56.5
Non-Hispanic Black	251	30.7
Non-Hispanic Chinese American	105	12.8
Education		
Less than high school	61	7.5
High school graduate/GED	56	6.9
College	397	48.6
Graduate school	303	37.1
Household income		
< 35 k	206	26.3
35 k - <75 k	196	25.1
75 k - <100 k	107	13.7
> = 100 k	273	34.9
Current working status		
Unemployed	326	39.9
Employed	491	60.1
Sidewalks in your neighborhood (yes)	807	98.7
How safe is neighborhood from crime		
1 - very safe	125	15.3
2	153	18.7
3 - safe	386	47.2
4	134	16.4
5 - not at all safe	20	2.4
Violence is a problem in neighborhood		
Very serious problem	15	1.8
Somewhat serious problem	99	12.1
Minor problem	298	36.4
Not really problem	406	49.6

Almost half of the participants reported their neighborhood to be safe from crime (level 3), with 34.0% reporting higher safety (level 1/2) and 18.7% reporting lower safety (level 4/5) (Table 1). Approximately half of participants reported that violence was not really a problem in their neighborhood, 49.6% reported that violence was a minor problem, and 13.9% reported that violence was a somewhat or very serious problem.

In the participant neighborhoods, reports of incivilities occurred most often, followed by criminal offenses and homicides (Table 2). A higher average proportion of the criminal offenses occurred outdoors (75.3%) when compared to the incivilities (42.0%). Spearman correlations between the three types of total normalized crimes occurring in the participant’s census block group ranged from 0.31 between incivilities and homicide (0.43 for outdoor crimes), 0.48 between criminal offenses and homicide (0.31 for outdoor crimes), and 0.81 between incivilities and criminal offenses (0.71 for outdoor crimes).

**Table 2:** Normalized Two-year Counts of Crimes Occurring in the Participant’s Census Block Group: The Multi-ethnic Study of Atherosclerosis

**Type of Crime**	**Location**	**Mean**	Normalized Crimes*	**Interquartile Range**
			**Median**	
Incivilities (drug, vandalism, prostitution)				
	Total	61.7	38.6	25.3 ~ 64.4
	Outdoor	25.1	16.1	10.5 ~ 27.0
Criminal offenses (robbery, weapon, sexual assault)				
	Total	15.0	10.8	4.7 ~ 18.1
	Outdoor	11.3	8.0	4.2 ~ 14.2
Homicide				
	Total	0.2	0.0	0 ~ 0
	Outdoor	0.1	0.0	0 ~ 0

*normalized crimes are defined as the number of crimes per 1000 population.

### Agreement between perceived safety and police-recorded crime

Agreement between perceived safety, assessed with the 2 questions, and police-recorded measures of crime was poor to fair (ranging from 0.10 to 0.28) using Spearman correlation coefficients, with no meaningful differences when comparing total crimes to outdoor crimes (data not shown). When considering the type of police-recorded crime, agreement between perceived safety and police-recorded crime was somewhat higher when using total or outdoor criminal offenses (range of correlations 0.19 to 0.28) as compared to incivilities (0.12 to 0.23) or homicides (0.10 to 0.20).

### Association of physical activity with perceived safety

The highest category of self-reported neighborhood safety (“very safe”) was associated with a greater odds of transport walking, while lower reported neighborhood violence (“not really a problem” or “minor problem”) was associated with a greater odds of leisure walking (Table 3). Participation in non-walking leisure activities was not associated with either of the perceived safety measures.

**Table 3:** Adjusted* Odds Ratios^ with 95% Confidence Intervals for Perception of Safety with Physical Activity: The Multi-ethnic Study of Atherosclerosis

**Perceived Safety**	Transport Walking**		Leisure Walking**		Non-walking Leisure Activities**	
	**Tertile 2 vs. 1**	**Tertile 3 vs. 1**	**Tertile 2 vs. 1**	**Tertile 3 vs. 1**	**Category 2 vs. 1**	**Category 3 vs. 1**
How safe is neighborhood from crime						
1 or 2 - very safe	1.38 (0.79,2.41)	1.72 (1.09,2.70)	1.07 (0.62,1.85)	1.54 (0.91,2.63)	0.87 (0.51,1.49)	1.11 (0.66,1.86)
3 - safe	1.11 (0.69,1.76)	1.45 (0.92,2.28)	1.15 (0.70,1.89)	1.33 (0.80,2.19)	0.94 (0.62,1.41)	0.97 (0.57,1.65)
4 or 5 - not safe	1.0	1.0	1.0	1.0	1.0	1.0
Violence is a problem in neighborhood						
Not really problem	1.09 (0.56,2.11)	0.78 (0.47,1.30)	1.48 (0.85,2.56)	1.99 (1.15,3.43)	0.99 (0.58,1.67)	0.97 (0.55,1.72)
Minor problem	1.04 (0.52,2.07)	0.99 (0.60,1.63)	1.18 (0.64,2.17)	1.88 (1.12,3.15)	1.18 (0.74,1.87)	1.09 (0.61,1.95)
Very/somewhat serious problem	1.0	1.0	1.0	1.0	1.0	1.0

^ Adjusted for clustering using 2000 census block groups by applying generalized estimating equations.

*All associations control for age (45-54, 55-64, 65-74, 75-84), gender, race/ethnicity (non-Hispanic White, non-Hispanic Black, non-Hispanic Chinese American), education (high school or less, college, graduate school), income (<35 k, 35 k- <75 k, 75 k- < 100 k, > = 100 k), working status (yes or no), sidewalk presence in neighborhood (yes or no), length of residence in neighborhood (continuous), and distance to nearest public transportation (continuous).

**Cutpoints of categorized physical activity outcomes in minutes/week:

Transport walking: (1) <150, (2) 150- < 420, (3) > =420.

Leisure walking: (1) <35, (2) 35- < 210, (3) > =210.

Non-walking leisure Activities: (1) 0, (2) <210, (3) > =210.

We explored whether the association between perceived safety and physical activity was differential with respect to gender, education, or race/ethnicity (data not shown), controlling for age, gender (except when stratified), education (except when stratified), race/ethnicity (except when stratified), working status, sidewalk presence, length of residence in neighborhood, and distance to public transportation. We consistently found that among women reporting that violence was not really a problem or that violence was a minor problem was associated with a higher odds of transport walking (odds ratio 1.80 (95% confidence interval (CI) 0.84, 3.84); 1.86 (95% CI 0.87, 4.01) respectively comparing the highest to the lowest category of walking). In contrast, men reporting that violence was not really a problem or was a minor problem was associated with a lower odds of transport walking (0.39 (95% CI 0.17, 0.92); 0.47 (95% CI 0.19, 1.16) respectively). Among women, reporting violence was not really a problem or a minor problem was also associated with a higher odds of leisure walking (2.77 (95% CI 1.31, 5.87); 2.25 (95% CI 1.07, 4.74) respectively comparing the highest to the lowest category of walking). In contrast, weaker and non-statistically significant associations were observed for transport walking among men (1.44 (95% CI 0.64, 3.24); 1.84 (0.78, 4.35) respectively). No interactions were observed for non-walking leisure activities.

### Association of physical activity with police-recorded crime

Table 4 shows adjusted associations of police-recorded crime measures with each of the three physical activity outcomes separately. The odds of being in the higher tertile (tertile 2 or 3) of transport walking were approximately 1.1 to 1.8 times higher among adults in the lowest tertile (1st) for total incivilities compared to those in the highest tertile (3rd). This association was stronger, ranging from 1.6 to 2.3 times higher, when exploring outdoor incivilities. Transport walking was not associated with total or outdoor criminal offenses.

**Table 4:** Adjusted* Odds Ratios^ with 95% Confidence Intervals for Normalized Measures of Crime with Physical Activity: The Multi-ethnic Study of Atherosclerosis

Police-Recorded Crime Measures***	Transport Walking**		Leisure Walking**		Non-walking Leisure Activities**	
	**Tertile 2 vs. 1**	**Tertile 3 vs. 1**	**Tertile 2 vs. 1**	**Tertile 3 vs. 1**	**Category 2 vs. 1**	**Category 3 vs. 1**
Incivilities (drug, vandalism, prostitution)						
Total						
1st tertile (lowest crime)	1.75 (1.12,2.72)	1.10 (0.69,1.76)	1.24 (0.80,1.90)	1.05 (0.70,1.59)	0.95 (0.58,1.57)	1.08 (0.66,1.75)
2nd tertile	1.13 (0.73,1.76)	0.94 (0.58,1.53)	1.06 (0.70,1.61)	1.15 (0.76,1.74)	1.41 (0.85,2.34)	1.42 (0.85,2.40)
3rd tertile (highest crime)	1.0	1.0	1.0	1.0	1.0	1.0
Outdoor						
1st tertile (lowest crime)	2.26 (1.50,3.42)	1.58 (1.01,2.49)	1.24 (0.80,1.93)	1.35 (0.88,2.06)	1.06 (0.63,1.77)	1.32 (0.85,2.03)
2nd tertile	1.36 (0.90,2.06)	1.05 (0.69,1.58)	1.10 (0.69,1.74)	0.90 (0.61,1.34)	0.86 (0.52,1.41)	0.93 (0.60,1.43)
3rd tertile (highest crime)	1.0	1.0	1.0	1.0	1.0	1.0
Criminal offenses (robbery, weapon, sexual assault)						
Total						
1st tertile (lowest crime)	1.44 (0.91,2.28)	1.14 (0.70,1.87)	0.84 (0.57,1.23)	0.96 (0.63,1.45)	0.82 (0.51,1.31)	1.37 (0.87,2.16)
2nd tertile	1.07 (0.68,1.70)	1.13 (0.77,1.65)	0.69 (0.44,1.07)	0.75 (0.47,1.20)	0.94 (0.60,1.48)	1.15 (0.75,1.77)
3rd tertile (highest crime)	1.0	1.0	1.0	1.0	1.0	1.0
Outdoor						
1st tertile (lowest crime)	1.14 (0.70,1.84)	1.14 (0.70,1.84)	0.75 (0.50,1.11)	0.76 (0.50,1.16)	0.80 (0.53,1.21)	1.22 (0.77,1.92)
2nd tertile	1.17 (0.76,1.81)	0.84 (0.54,1.32)	0.68 (0.43,1.09)	0.62 (0.39,0.96)	1.15 (0.73,1.82)	1.32 (0.86,2.01)
3rd tertile (highest crime)	1.0	1.0	1.0	1.0	1.0	1.0
Homicide						
Total						
none	0.90 (0.55,1.48)	0.82 (0.53,1.29)	0.88 (0.54,1.43)	0.91 (0.54,1.52)	0.82 (0.42,1.58)	1.02 (0.62,1.67)
yes (< median of non-zero data)	0.95 (0.43,2.06)	0.48 (0.22,1.04)	0.80 (0.36,1.76)	0.88 (0.36,2.11)	0.50 (0.19,1.34)	0.59 (0.29,1.20)
yes (> = median of non-zero data)	1.0	1.0	1.0	1.0	1.0	1.0
Outdoor						
none	0.97 (0.53,1.80)	1.12 (0.68,1.86)	1.08 (0.53,2.19)	0.99 (0.52,1.87)	0.72 (0.38,1.38)	1.39 (0.73,2.62)
yes (< median of non-zero data)	0.94 (0.35,2.48)	0.63 (0.25,1.59)	1.04 (0.36,3.01)	1.05 (0.36,3.07)	0.80 (0.30,2.18)	0.95 (0.31,2.90)
yes (> = median of non-zero data)	1.0	1.0	1.0	1.0	1.0	1.0

^ Adjusted for clustering using 2000 census block groups by applying generalized estimating equations.

*All associations control for age (45-54, 55-64, 65-74, 75-84), gender, race/ethnicity (non-Hispanic White, non-Hispanic Black, non-Hispanic Chinese American), education (high school or less, college, graduate school), income (<35 k, 35 k- < 75 k, 75 k- < 100 k, > = 100 k), working status (yes or no), sidewalk presence in neighborhood (yes or no), length of residence in neighborhood (continuous), and distance to nearest public transportation (continuous).

**Cutpoints of categorized physical activity outcomes in minutes/week:

Transport walking: (1) <150, (2) 150- < 420, (3) > =420.

Leisure walking: (1) <35, (2) 35- < 210, (3) > =210.

Non-walking Leisure Activities: (1) 0, (2) <210, (3) > =210.

***Normalized crimes were summarized over two years, which include the year the interview was performed and the previous year.

Cutpoints of categorized crime measures in tertiles:

Criminal offenses: Total: (1) <6.2, (2) 6.2- < 15.2, (3) > =15.2; Outdoor: (1) <4.8, (2) 4.8- < 11.6, (3) > =11.6.

Incivilities: Total: (1) <27.8, (2) 27.8- < 54.6, (3) > =54.6; Outdoor: (1) <12.9, (2) 12.9- < 22.7, (3) > =22.7.

Homicides: Total: (1) 0, (2) >0- < 0.9, (3) > =0.9; Outdoor: (1) 0, (2) >0- < 0.9, (3) > =0.9.

Leisure walking was not associated with total incivilities, outdoor incivilities, or total criminal offenses. The odds of being in the higher tertiles (2nd and 3rd) of leisure walking was lower among adults in the lower two tertiles (1st and 2nd) for outdoor criminal offenses compared to those in the highest (3rd) tertile. Transport walking and leisure walking were not associated with total or outdoor homicides. Participation in non-walking leisure activities was not associated with any of the police-recorded crime measures.

### Association of physical activity with both perceived safety and police-recorded crime

#### Independent associations

The addition of police-recorded measures of crime to the models with the perceived measures resulted in better model fit according to the likelihood ratio tests for all 12 models (Table 5). Results for non-walking leisure activities are not shown, but did not result in any significant associations.

**Table 5:** Adjusted* Odds Ratios with 95% Confidence Intervals from Models that Include Both Perceived Safety and Police-Recorded Crimes with Physical Activity: The Multi-ethnic Study of Atherosclerosis

**Model**	Exposure*	Transport Walking**		Leisure Walking**	
		**Tertile 2 vs. 1**	**Tertile 3 vs. 1**	**Tertile 2 vs. 1**	**Tertile 3 vs. 1**
1a	How safe is neighborhood from crime				
	1 or 2 - very safe	1.28 (0.72,2.27)	1.70 (1.08,2.68)	1.04 (0.59,1.83)	1.53 (0.90,2.61)
	3 - safe	1.06 (0.66,1.69)	1.45 (0.92,2.27)	1.14 (0.69,1.86)	1.32 (0.80,2.20)
	4 or 5 - not safe	1.0	1.0	1.0	1.0
	Incivilities(total) ***				
	1st tertile (lowest crime)	1.72 (1.10,2.69)	1.09 (0.68,1.76)	1.24 (0.80,1.92)	1.00 (0.66,1.53)
	2nd tertile	1.14 (0.73,1.78)	0.95 (0.58,1.54)	1.07 (0.70,1.63)	1.11 (0.74,1.66)
	3rd tertile (highest crime)	1.0	1.0	1.0	1.0
1b	How safe is neighborhood from crime				
	1 or 2 - very safe	1.31 (0.74,2.32)	1.70 (1.07,2.72)	1.09 (0.62,1.90)	1.55 (0.92,2.64)
	3 - safe	1.06 (0.67,1.70)	1.44 (0.90,2.29)	1.15 (0.70,1.88)	1.34 (0.81,2.20)
	4 or 5 - not safe	1.0	1.0	1.0	1.0
	Criminal offenses (total) ***				
	1st tertile (lowest crime)	1.38 (0.86,2.20)	1.10 (0.66,1.84)	0.83 (0.56,1.24)	0.88 (0.57,1.35)
	2nd tertile	1.05 (0.66,1.68)	1.11 (0.75,1.63)	0.69 (0.44,1.08)	0.74 (0.46,1.19)
	3rd tertile (highest crime)	1.0	1.0	1.0	1.0
1c	How safe is neighborhood from crime				
	1 or 2 - very safe	1.41 (0.80,2.49)	1.73 (1.08,2.76)	1.09 (0.62,1.90)	1.57 (0.90,2.76)
	3 - safe	1.11 (0.70,1.78)	1.49 (0.93,2.37)	1.17 (0.71,1.92)	1.33 (0.80,2.21)
	4 or 5 - not safe	1.0	1.0	1.0	1.0
	Homicide (total) ***				
	none	0.86 (0.52,1.43)	0.77 (0.49,1.20)	0.87 (0.53,1.43)	0.85 (0.50,1.46)
	yes (<median of none zero data)	0.91 (0.42,1.97)	0.47 (0.21,1.03)	0.78 (0.35,1.72)	0.89 (0.36,2.18)
	yes (> = median of none zero data)	1.0	1.0	1.0	1.0
2a	Violence is a problem in neighborhood				
	Not really problem	1.02 (0.52,2.00)	0.78 (0.47,1.29)	1.44 (0.83,2.50)	1.98 (1.15,3.42)
	Minor problem	1.00 (0.50,1.99)	1.00 (0.60,1.67)	1.17 (0.63,2.16)	1.88 (1.12,3.17)
	Somewhat/Very serious problem	1.0	1.0	1.0	1.0
	Incivilities (total) ***				
	1st tertile (lowest crime)	1.75 (1.12,2.72)	1.12 (0.70,1.80)	1.18 (0.77,1.83)	1.02 (0.66,1.55)
	2nd tertile	1.14 (0.73,1.78)	0.92 (0.56,1.51)	1.05 (0.69,1.60)	1.12 (0.75,1.67)
	3rd tertile (highest crime)	1.0	1.0	1.0	1.0
2b	Violence is a problem in neighborhood				
	Not really problem	1.07 (0.55,2.07)	0.79 (0.47,1.31)	1.50 (0.87,2.57)	2.00 (1.17,3.42)
	Minor problem	1.00 (0.51,1.97)	1.00 (0.60,1.65)	1.16 (0.63,2.13)	1.89 (1.13,3.15)
	Somewhat/Very serious problem	1.0	1.0	1.0	1.0
	Criminal offenses (total) ***				
	1st tertile (lowest crime)	1.44 (0.91,2.28)	1.16 (0.70,1.92)	0.79 (0.54,1.16)	0.91 (0.59,1.38)
	2nd tertile	1.07 (0.68,1.69)	1.14 (0.78,1.67)	0.68 (0.44,1.05)	0.74 (0.46,1.19)
	3rd tertile (highest crime)	1.0	1.0	1.0	1.0
2c	Violence is a problem in neighborhood				
	Not really problem	1.10 (0.56,2.16)	0.78 (0.46,1.31)	1.48 (0.85,2.57)	2.00 (1.15,3.47)
	Minor problem	1.04 (0.52,2.09)	0.99 (0.59,1.64)	1.18 (0.64,2.17)	1.88 (1.12,3.14)
	Somewhat/Very serious problem	1.0	1.0	1.0	1.0
	Homicide (total) ***				
	none	0.90 (0.54,1.49)	0.85 (0.54,1.34)	0.86 (0.52,1.40)	0.89 (0.53,1.50)
	yes (<median of none zero data)	0.95 (0.44,2.06)	0.48 (0.22,1.05)	0.82 (0.37,1.80)	0.90 (0.36,2.27)
	yes (> = median of none zero data)	1.0	1.0	1.0	1.0

^ Adjusted for clustering using 2000 census block groups by applying generalized estimating equations.

*All associations control for age (45-54, 55-64, 65-74, 75-84), gender, race/ethnicity (non-Hispanic White, non-Hispanic Black, non-Hispanic.

Chinese American), education (high school or less, college, graduate school), income (<35 k, 35 k- < 75 k, 75 k- < 100 k, > = 100 k), working status (yes or no), sidewalk presence in neighborhood (yes or no), length of residence in neighborhood (continuous), and distance to nearest public transportation (continuous).

**Cutpoints of categorized physical activity outcomes in minutes/week:

Transport walking: (1) <150, (2) 150- < 420, (3) > =420.

Leisure walking: (1) <35, (2) 35- < 210, (3) > =210.

***Objectively measured normalized crimes were summarized over two years, which include the year the interview was performed and the previous year.

Criminal offenses: Total: (1) <6.2, (2) 6.2- < 15.2, (3) > =15.2; Outdoor: (1) <4.8, (2) 4.8- < 11.6, (3) > =11.6.

Incivilities: Total: (1) <27.8, (2) 27.8- < 54.6, (3) > =54.6; Outdoor: (1) <12.9, (2) 12.9- < 22.7, (3) > =22.7.

Homicides: Total: (1) 0, (2) >0- < 0.9, (3) > =0.9; Outdoor: (1) 0, (2) >0- < 0.9, (3) > =0.9.

#### Combined associations

We explored the association of combinations of perceived safety (i.e., how safe is your neighborhood from crime) and police-recorded crime with physical activity (Table 6). The adjusted odds of being in the higher tertiles (2nd or 3rd) for transport walking was 2.2 to 2.4 times higher among adults both reporting a very safe neighborhood and being in the lowest tertile (1st) for outdoor incivilities (low perceived and police-recorded crime) compared to those both reporting unsafe neighborhoods and having the highest tertile (3rd) of outdoor incivilities in their neighborhood (high perceived and police-recorded crime). Weaker associations were identified when using criminal offenses and perceived safety for the transport walking outcome. Transport walking was not associated with combined safety measures using total homicides. Leisure walking and non-walking leisure activities (data not shown) were not significantly associated with any combined safety measures.

**Table 6:** Adjusted* Odds Ratios^ with 95% Confidence Intervals for Combined Perceived Safety and Police-Recorded Crime with Physical Activity: The Multi-ethnic Study of Atherosclerosis

**Perceived Safety / Police Recorded**	**n**	**%**	Transport Walking**		Leisure Walking**	
**Measure of Crime**			**Tertile 2 vs. 1**	**Tertile 3 vs. 1**	**Tertile 2 vs. 1**	**Tertile 3 vs. 1**
How safe is your neighborhood from crime?						
Incivilities (total)						
1 or 2 - very safe/lowest tertile	98	12.3	1.98 (0.97,4.02)	1.56 (0.81,3.03)	1.10 (0.49,2.47)	1.01 (0.50,2.03)
Other	628	78.9	1.04 (0.62,1.75)	1.21 (0.74,1.97)	1.20 (0.63,2.26)	1.23 (0.70,2.16)
4 or 5 -not safe/highest tertile	70	8.8	1.0	1.0	1.0	1.0
Incivilities (outdoor)						
1 or 2 - very safe/lowest tertiles	128	16.1	2.42 (1.18,4.98)	2.19 (1.15,4.16)	1.04 (0.49,2.21)	1.41 (0.69,2.91)
Other	601	75.5	1.18 (0.69,2.01)	1.33 (0.82,2.14)	1.04 (0.55,1.95)	1.17 (0.67,2.04)
4 or 5 -not safe/highest tertiles	67	8.4	1.0	1.0	1.0	1.0
Criminal offenses (total)						
1 or 2 - very safe/lowest tertile	128	16.1	1.95 (1.03,3.70)	1.61 (0.89,2.88)	0.77 (0.36,1.65)	0.91 (0.43,1.89)
Other	598	75.1	1.11 (0.65,1.87)	1.42 (0.90,2.27)	0.85 (0.47,1.53)	0.79 (0.42,1.46)
4 or 5 - not safe/highest tertile	70	8.8				
Criminal offenses (outdoor)						
1 or 2 - very safe/lowest tertiles	129	16.2	1.68 (0.82,3.46)	1.46 (0.80,2.65)	0.78 (0.37,1.66)	0.77 (0.36,1.62)
Other	593	74.5	1.03 (0.63,1.67)	1.26 (0.80,1.98)	0.86 (0.48,1.54)	0.81 (0.45,1.45)
4 or 5 - not safe/highest tertiles	74	9.3	1.0	1.0	1.0	1.0
Safety: Homcide (total)						
1 or 2 - very safe/lowest tertile	253	31.8	1.01 (0.45,2.27)	1.67 (0.85,3.26)	1.24 (0.47,3.25)	1.34 (0.65,2.79)
Other	520	65.3	0.83 (0.36,1.90)	1.33 (0.68,2.60)	1.28 (0.49,3.34)	1.07 (0.47,2.43)
4 or 5 - not safe/highest tertile	23	2.9	1.0	1.0	1.0	1.0

^ Adjusted for clustering using 2000 census block groups by applying generalized estimating equations.

*All associations control for age (45-54, 55-64, 65-74, 75-84), gender, race/ethnicity (non-Hispanic White, non-Hispanic Black, non-Hispanic Chinese American), education (high school or less, college, graduate school), income (<35 k, 35 k- < 75 k, 75 k- < 100 k, > = 100 k), working status (yes or no), sidewalk presence in neighborhood (yes or no), length of residence in neighborhood (continuous), and distance to nearest public transportation (continuous).

**Cutpoints of categorized physical activity outcomes in minutes/week:

Transport walking: (1) <150, (2) 150- < 420, (3) > =420.

Leisure walking: (1) <35, (2) 35- < 210, (3) > =210.

Note: associations with outdoor homicides could not be conducted due to small cell sizes.

We explored whether the associations in these models was modified by gender, education, or race/ethnicity. Gender was an effect modifier, with stronger positive associations between a safer neighborhood (defined by both perceived and police-reported measures) and more transport walking among women but not men, using total or outdoor police-recorded assessment of criminal offenses.

## Discussion

In this study of diverse urban residents, several perceived safety and police-recorded crime measures were associated with transport and leisure walking, but not with non-walking leisure activity. This may be because some of these sport activities are not engaged in outdoors or within the neighborhood. Alternatively, non-walking leisure activities may be less affected by crime and perceived safety. A comprehensive review of studies [1] concluded that there was insufficient evidence to determine that safety was associated with physical activity. However, many of these reviewed studies had limited measures of safety and often did not explore both perceived and more objective measures of safety. Since the review, several other studies explored perceived measures of safety (for example [21-26]), others explored police-recorded measures of crime (for example [27,28]), and three studies explored both perceived and objective safety measures [5,11,12]. Most of these more recent studies identified associations of safety or crime with physical activity. Similar to our findings, prior studies found low agreement between perceived and objective measures of safety and crime, regardless of how measured, suggesting that the two measures might be assessing different dimensions of one’s physical environment [5,9,11,12].

Our results on transportation walking are consistent with other work documenting that fear of crime affects use of public transportation [29]. For example, studies have documented fear of crime at transit stations and several researchers [30,31] argue that fear of crime has an impact on travel decisions by individuals. Analysis of the environs of rapid transit stations in Chicago indicated that areas near a transit station in relatively low crime neighborhoods had higher levels of crime than the surrounding area [32,33]. We demonstrated that crime was associated with a lower probability of transport walking. Thus, strategies to reduce crime in neighborhoods and around public transportation areas may not only increase the use of public transportation, with consequent environmental benefits, but may also increase physical activity through the promotion of walking.

Our results confirm some aspects of the frameworks proposed by others [1,2], in that both perceived safety and police-recorded measures of crime appear to be independently associated with physical activity, in particular walking. While police-recorded crime in the neighborhood was only weakly correlated with perceived safety, similar to other studies [5,9,11,12,28,34,35], it also was an independent predictor of transport walking or exercise. Furthermore, the strongest associations between safety and transport walking and exercise were observed among persons living in the safest neighborhoods, according to the combined measures of safety that utilized both reporting a safe neighborhood and police-recorded measures of crime as compared to those living in areas that both were reported as being unsafe and fell in the highest category for police-recorded crimes. To explore these combined associations of both perceived and police-recorded crime, we created variables that grouped the safest and unsafest groups together, with a third category of all other participants. With more data, an alternative exploration could be made into participants that over-reported their safety (participant perceives neighborhood is safe but police reports indicate it is unsafe) and under-reported their safety (participant perceives neighborhood is unsafe but police reports indicate it is safe).

### Interactions

We found that among women perception of safety was strongly associated with transport walking or leisure in a variety of models, while the associations were weaker or null among men. This is in line with a US study of adults living in low-income housing that found that a perception of feeling unsafe at night was associated with fewer pedometer recorded steps/day among women but not men [21]. Another study of 75–76 year old Norwegian women and men found that violence in the neighborhood was associated with physical activity among men, while perceived safety was associated with physical activity among women [12]. We did not observe interactions of perceived safety with education. This is in agreement with a US study of adults age 50 years and older [25]. We also did not identify interactions of perceived safety with race/ethnicity. Other studies are needed with larger sample sizes and diverse populations to further explore these associations.

### Limitations

Our study has several important limitations. First, other confounders may exist that we did not account for in the adjusted models. For example, we were unable to control for self-selection to the neighborhood (i.e., if a participant lives in a neighborhood because it is safer to walk), nor did we assess for their actual victimization experience. Second, in this exploratory study we have tested many associations, but did not adjust for multiple testing. Therefore, significance should be interpreted with caution, and replication of results is needed. It should be noted that the findings were attenuated when adjusting for the neighborhood correlation, indicating the influence the correlation had on the findings. Third, while the study assessed self-reported physical activity comprehensively, a supplemental objective measure of physical activity was not assessed. Moveover, the prevalence of physical activity was likely over-reported because of the number of activities queried upon. Due to self-report bias and the skewness of the measures, physical activity was categorized, which resulted in some loss of precision. Fourth, we also have limited information on where people engaged in physical activity, although transport walking most likely occurs near the home or workplace. The baseline survey indicated that 68.3% of these Chicago MESA participants reported that the place they use most often for exercise was 1 mile or less from their home. Additionally, 90.8% reported exercising within a 20-minute walk or 1 mile from their home.

Fifth, the detailed crime data was summarized to the census block group level so there may be some misattribution. Although the census block group represents a small area, especially in this dense location, it still may not adequately represent the neighborhood of the participant. Data were not available at the time the analyses were conducted to examine crime measures for other spatial contexts, such as distance around home or participant-defined boundaries. Little theory or prior data exists on which to base the relevant definition of neighborhood for these analyses [36]. Moreover, the reported crimes are likely to be under-reported. There may also be some mismatch in time between the general recall of neighborhood safety as compared to the 2-year period of police-recorded crimes. We chose a 2-year period to reflect more stable trends in the area, but other windows of time may be more appropriate. Sixth, for the self-reported safety measures, both items lacked sufficient sample at the extreme unsafe range (not at all safe and violence a serious problem), such that these categories were collapsed. Seventh, this study was limited by its cross-sectional design. Finally, generalizability may be limited as this study focused on one Midwestern urban city in the US among 45 to 84 year old adults. Moreover, we were unable to explore the possibility of selection bias with our sample.

### Future studies

As noted earlier, the literature indicates that the relationship between physical activity and safety may be differential by gender, socioeconomic status, and race/ethnicity [4]. In addition, these relationships may be modified by psychosocial or environmental factors. Further in-depth qualitative and quantitative studies with sufficient statistical power and diversity should explore these complex relationships. Moreover, the inclusion of other age groups would be worthwhile, as this study focused on 45 to 84 year old adults.

While difficult to explore with epidemiologic studies, an exploration of the microenvironments that provide proximate cues regarding safety is also needed to understand how these features influence the choice to walk or be active in these settings [37,38]. Examples of features that might influence use of a space include prospect (is the view blocked or open), refuge (is there a hiding place), escape (open or blocked exit), naturalness (natural or developed elements), upkeep (maintenance), openness (visual scope or vista), number of people, type of land use (business, park, etc.), and complexity (amount of different noticeable elements) [38].

While we were able to explore aspects of the frameworks proposed by others [1,2], greater specificity of measurement and comprehensiveness continue to be needed. Further, safety may mediate the relationship between these environmental features and physical activity. As proposed by others [2], the relationship between perceived safety and physical activity may be bidirectional, with perceived safety influencing physical activity and also physical activity behavior influencing perceived safety. Longitudinal studies would enhance this work by helping with the temporality of the associations. Interventions are also needed to target groups with the highest perception of crime and in the areas with the highest objective measures of crime to potentially have the greatest population impact not only on physical activity but on other measures (e.g., general health, stress, neighborhood collective efficacy).

## Conclusion

Using a population-based study we showed that some perceived safety and police reported measures of crime were associated with both transport and leisure walking in a sample of urban adults. With regards to the police-recorded measures of crime, this study brought greater specificity by exploring type of crime (e.g., incivilities, criminal offenses, homicides) and whether crime occurred outdoors or not. Our results demonstrate the need for specificity, as called for by others [13], with regards to both exposure and outcome measures, and exploration into the gender differences in these relationships. The work reported here provides support for continuing to explore the role of perceived and objective measures of safety on transport or leisure walking. Since walking may be an important source of physical activity, greater attention to reductions of violence and crime and increased perceptions of neighborhood safety may contribute to higher population levels of physical activity.

## Abbreviations

CI: Confidence interval; GIS: Geographic Information Systems; IQR: Interquartile range; MESA: Multi-ethnic Study of Atherosclerosis; US: United States.

## Competing interests

The authors declare that they have no competing interests.

## Authors’ contributions

KRE oversaw the development of the study, the acquisition of data, and drafted the article. RB provided the police-recorded crime measures and FW conducted the statistical analysis. AVDR, APM, and DAR assisted with the design of the study. All authors critically reviewed the article several times and gave final approval for its submission.

## References

[B1] Foster S Giles-Corti B The built environment, neighborhood crime and constrained physical activity: An exploration of inconsistent findings Prev Med 2008 47 3 241 251 10.1016/j.ypmed.2008.03.017 18499242

[B2] Loukaitou-Sideris A Eck J Crime prevention and active living Am J Health Promotion 2007 21 4 supplement 380 389 10.4278/0890-1171-21.4s.380 17465184

[B3] Day K Active living and social justice J Am Plan Assoc 2006 72 1 88 99 10.1080/01944360608976726

[B4] Loukaitou-Sideris A Is it safe to walk? Neighborhood safety and security considerations and their effects on walking J Plan Lit 2006 20 3 219 232 10.1177/0885412205282770

[B5] McGinn A Evenson K Herring A Huston S Rodriguez D The association of perceived and objectively measured crime with physical activity: A cross-sectional analysis J Phys Act Health 2008 5 1 117 131 18209258 10.1123/jpah.5.1.117 PMC4950861

[B6] Block R Block C Decisions and data: The transformation of robbery incidents into robbery statistics J Criminal Law Criminology 1980 71 4 622 636 10.2307/1142828

[B7] Brownstein H The social production of crime statistics Justice Res Policy 2000 2 2 73 90 10.3818/JRP.2.2.2000.73

[B8] Mosher C Miethe T Phillips D The Mismeasure of Crime 2002 Thousand Oaks, CA: Sage Publications

[B9] Kirtland K Porter D Addy C Neet M Williams J Sharpe P Neff L Kimsey C Jr Ainsworth B Environmental measures of physical activity supports: perception versus reality Am J Prev Med 2003 24 4 323 331 10.1016/S0749-3797(03)00021-7 12726870

[B10] Wilson D Kirtland K Ainsworth B Addy C Socioeconomic status and perception of access and safety for physical activity Ann Behavior Med 2004 28 1 20 28 10.1207/s15324796abm2801_4 15249256

[B11] Oh AY Zenk SN Wilbur J Block R McDevitt J Wang E Effects of perceived and objective neighborhood crime on walking frequency among midlife African American women in a home-based walking intervention J Phys Act Health 2010 7 4 432 441 20683084 10.1123/jpah.7.4.432

[B12] Piro F Oyvind N Claussen B Physical activity among elderly people in a city population: the influence of neighborhood level violence and self perceived safety J Epidemiol Comm Health 2006 60 626 632 10.1136/jech.2005.042697 PMC2566241 16790836

[B13] Giles-Corti B Timperio A Bull F Pikora T Understanding physical activity environmental correlates: increased specificity for ecological models Exercise Sport Sci Rev 2005 33 4 175 181 10.1097/00003677-200510000-00005 16239834

[B14] Bild D Bluemke D Burke G Detrano R Diez Roux A Folsom A Greenland P Jacobs D Jr Kronmal R Liu K Multi-ethnic Study of Atherosclerosis: objectives and design Am J Epidemiol 2002 156 9 871 881 10.1093/aje/kwf113 12397006

[B15] Ainsworth B Irwin M Addy C Whitt M Stolarczyk L Moderate physical activity patterns of minority women: the cross-cultural activity participation study J Women Health Gender Based Med 1999 8 6 805 813 10.1089/152460999319129 10495261

[B16] LaMonte M Durstine J Addy C Irwin M Ainsworth B Physical activity, physical fitness, and Framingham 10-year risk score: the cross-cultural activity participation study J Cardiopulm Rehabil 2001 21 63 70 10.1097/00008483-200103000-00001 11314285

[B17] Henderson K Ainsworth B A synthesis of perceptions about physical activity among older African American and American Indian women Am J Public Health 2003 93 2 313 317 10.2105/AJPH.93.2.313 12554592 PMC1447736

[B18] Patterson P Reliability, validity, and methodological response to the assessment of physical activity via self-report Research Q Exercise Sport 2000 71 15 20 10.1080/02701367.2000.11082781 25680008

[B19] Echeverria S Diez Roux A Link B Reliability of self-reported neighborhood characteristics J Urban Health 2004 81 4 682 701 10.1093/jurban/jth151 15466849 PMC3455923

[B20] Mujahid MS Diez Roux AV Morenoff JD Raghunathan T Assessing the measurement properties of neighborhood scales: from psychometrics to ecometrics Am J Epidemiol 2007 165 8 858 867 10.1093/aje/kwm040 17329713

[B21] Bennett GG McNeill LH Wolin KY Duncan DT Puleo E Emmons KM Safe to walk? Neighborhood safety and physical activity among public housing residents PLoS Med 2007 4 10 1599 1607 17958465 10.1371/journal.pmed.0040306 PMC2039759

[B22] Pichon LC Arredondo EM Roesch S Sallis JF Ayala GX Elder JP The relation of acculturation to Latinas’ perceived neighborhood safety and physical activity: a structural equation analysis Ann Behav Med 2007 34 3 295 303 10.1007/BF02874554 18020939

[B23] Sallis JF King AC Sirard JR Albright CL Perceived environmental predictors of physical activity over 6 months in adults: activity counseling trial Health Psych 2007 26 6 701 709 10.1037/0278-6133.26.6.701 18020842

[B24] King D Neighborhood and individual factors in activity in older adults: results from the neighborhood and senior health study J Aging Phys Act 2008 16 2 144 170 18483439 10.1123/japa.16.2.144

[B25] Tucker-Seeley RD Subramanian SV Li Y Sorensen G Neighborhood safety, socioeconomic status, and physical activity in older adults Am J Prev Med 2009 37 3 207 213 10.1016/j.amepre.2009.06.005 19595554 PMC3685411

[B26] Kamphuis CB Mackenbach JP Giskes K Huisman M Brug J van Lenthe FJ Why do poor people perceive poor neighbourhoods? The role of objective neighbourhood features and psychosocial factors Health Place 2010 16 4 744 754 10.1016/j.healthplace.2010.03.006 20382555

[B27] McDonald N The effect of objectively measured crime on walking in minority adults Am J Health Promot 2008 22 6 433 436 10.4278/ajhp.22.6.433 18677884

[B28] Roman C Chalfin A Fear of walking outdoors: A multilevel ecologic analysis of crime and disorder Am J Prev Med 2008 34 4 306 312 10.1016/j.amepre.2008.01.017 18374244

[B29] Brantingham P Brantingham P Criminality of place: Crime generators and crime attractors Eur J Criminal Policy Res 1995 3 5 26

[B30] Kenney D Crime, Fear, and the New York City Subway 1987 New York: Praeger

[B31] Loukaitou-Sideris A Liggett R Iseki H The geography of transit crime: Documentation and evaluation of crime incidence on and around the green line stations in Los Angeles J Plann Educ Res 2002 22 135 151 10.1177/0739456X02238443

[B32] Block R Davis S Clarke R The environs of rapid transit stations: a focus for street crime or just another risky place? Preventing Mass Transit Crime (Crime Prevention Studies, vol 6) 1996 Monsey, NY: Criminal Justice Press 238 257

[B33] Block R Block C Goldsmith V, McGuire P, Mollenkopf J, Ross T The Bronx and Chicago: street robbery in the environs of rapid transit stations Analyzing Crime Patterns: Frontiers of Practice 2000 Thousand Oaks, CA: Sage Publishing 137 152

[B34] Taylor R Gottfredson S Brower S Block crime and fear: defensible space, local social ties, and territorial functioning J Research Crime Delinquency 1984 21 303 331 10.1177/0022427884021004003

[B35] Skogan W Disorder and Decline: Crime and the Spiral Decay in American Neighborhoods 1990 Toronto: Free Press

[B36] Weisburd D Bruinsma G Bernasco W Putting Crime in Its Place: Units of Analysis in Geographic Criminology New York City, 2009 New York City, NY: Springer Science and Business Media

[B37] Nasar J Fisher B “Hot spots” of fear of crime: a multiple-method investigation J Environ Psych 1993 13 187 206 10.1016/S0272-4944(05)80173-2

[B38] Nasar J Assessing perceptions of environments for active living Am J Prev Med 2008 34 4 357 363 10.1016/j.amepre.2008.01.013 18374252

